# A global perspective on autoinducer-2-mediated cell communication in prokaryotes

**DOI:** 10.1016/j.isci.2025.112908

**Published:** 2025-06-13

**Authors:** Xiaozhen Liu, Zhiyan Wei, Mingming Yang, Xiaoxue Zhang, Zhuo Wang, Shuyu Li, Changfu Li, Lingfang Zhu, Lei Zhang, Xiaoqing Zhang, Xihui Shen

**Affiliations:** 1 Shaanxi Key Laboratory of Agricultural and Environmental Microbiology, College of Life Science, Northwest A&F University, Yangling, Shaanxi 712100, China; 2 Institute of Grassland Research, Chinese Academy of Agricultural Sciences, Hohhot 010010, China; 3 College of Plant Protection, Northwest A&F University, Yangling 712100, China; 4 Shanxi Key Laboratory of Yuncheng Salt Lake Ecological Protection and Resource Utilization, College of Life Sciences, Yuncheng University, Yuncheng, Shanxi 044000, China

**Keywords:** Biological sciences, Molecular biology, Microbiology

## Abstract

Animal communication within a community is essential for their survival and reproduction. This phenomenon is not restricted to animals but is also prevalent in prokaryotes, which employ quorum sensing (QS) mechanisms for communication and behavior. Despite their importance, cell-cell communication in prokaryotes has not been cataloged in detail. Therefore, we developed a comprehensive map of cell-cell microbial communication by analyzing 15,297 prokaryotic genomes, thereby expanding the scope of the AI-2 signal in the microbiome. We showed that LsrB receptors are found in 15 bacterial phyla and can regulate the expression of genes involved in the biosynthesis of siderophore group nonribosomal peptides and biofilm formation via LsrB in *Streptomyces coelicolor*. We also conducted an international prospective study of microbial communication models across distinct habitats. These findings highlight the importance of microbial communication and will enable comprehensive studies of large-scale microbial communities, greatly expanding our understanding of AI-2 in the microbial consortium.

## Highlights

• A genomic approach reveals the comprehensive map of AI-2-mediated prokaryotic communication
• Many bacteria recognize AI-2 via distinct receptors to affect population-level functions
• Cell communications are universal in different habitats, but with distinct modes

## Graphical abstract

**Figure:**
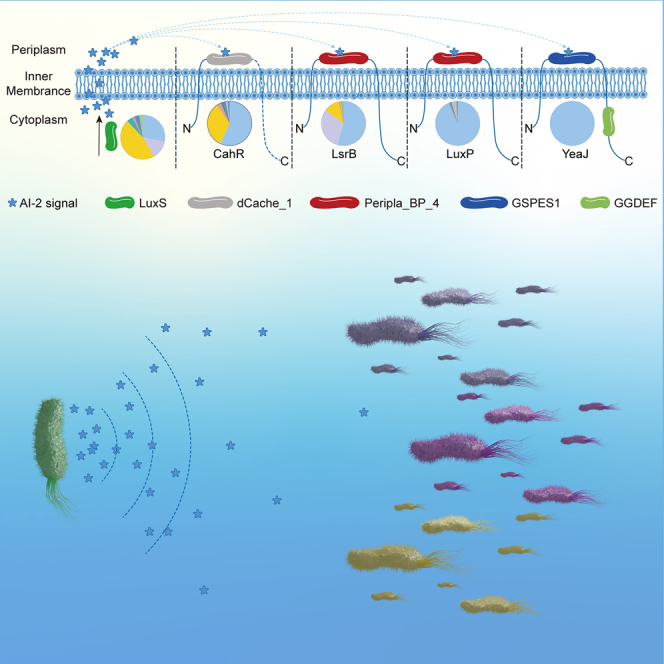

## Introduction

Microbial communities are present in virtually every ecological niche, including oceans,^1^ soils,^2^ plants,^3^ and animals.^4^ These communities drive the biogeochemical cycles,^5^ and global nutrient cycling,^6^ and influence plant and animal health.^3^^,^^4^^,^^7^ As microbes evolved, they developed multiple mechanisms to sense, communicate, and compete with other microbes. Quorum sensing (QS) is a cell-cell communication process that is mediated through the synthesis and release of autoinducers, such as acyl-homoserine lactone, autoinducing peptides, and autoinducer-2 (AI-2). Thus, QS regulates gene expression and controls collective behaviors among microbes in a cell density-dependent manner.^8^^,^^9^ Recent studies have examined the role of QS in microbe-host interactions. The QS system acts as an interkingdom signaling system,^10^ assisting in microbial colonization and virulence within the host.^11^ Therefore, understanding the processes underlying cell-cell communications and their social effects is crucial for predicting the potential impacts of microbes on ecosystems worldwide.

AI-2 is a universal QS signal that is produced and recognized by many bacteria and may play important roles in natural consortia and microbiomes.^12^ Mediation of the QS system by AI-2 enables bacteria to modify a wide variety of phenotypic characteristics. For example, AI-2 inhibits colonization by *Vibrio cholerae*,^13^ and affects chemotaxis, autoaggregation, biofilm formation, and stress resistance in *Escherichia coli*^14^ as well as contributes to the reestablishment of healthy gut microbial populations by favoring the expansion of firmicutes over bacteroidetes,^12^ and may help to degrade plant feedstuffs within the rumen.^15^ Specifically, AI-2 is synthesized from S-ribosylhomocysteine (SRH) by S-ribosylhomocysteinase (LuxS), as a byproduct of the activated methyl cycle in cells,^11^ after which it accumulates in the extracellular space and is recognized by specific receptors. LuxP AI-2 receptors were first identified in *Vibrio* spp.^16^ and LsrB in enteric bacteria^17^ and other microbes, such as those in families Clostridiaceae, Rhizobiaceae and Bacillaceae,^18^^,^^19^ however, large numbers of bacteria lacking LuxP or LsrB receptors can respond to AI-2. Due to the lack of information on AI-2 receptors, it remains challenging to understand how microbes respond to AI-2. Our research team recently discovered two novel AI-2 receptor types: including CahR^20^ and YeaJ.^21^ CahR-type receptors represent a class of extracytoplasmic sensors of signal transduction proteins,^20^ such as methyl-accepting chemotaxis proteins (MCPs), c-di-GMP-cyclases and diesterases (GCDs), serine phosphatases (SPs), histidine kinases (HKs), adenylate- and guanylate cyclases (AC/GCs), serine/threonine kinases (STKs).^22^ AI-2 binds to and activates the dCache_1 domains of CahR-type receptors to regulate the expression of corresponding genes, which subsequently cause the activation of downstream signaling pathways to modify social behaviors. For example, AI-2 modifies biofilm formation and chemotaxis of *Pseudomonas aeruginosa* by binding to the dCache_1 domain of CahR-type chemoreceptors.^20^ In addition, AI-2 binds to and activates the GAPES1 domain of *Salmonella enterica* serovar Typhimurium repress YeaJ-type receptors to induce c-di-GMP synthesis to repress T3SS-1 gene expression.^21^ However, there has been very little empirical research on AI-2 sensors within natural environments, limiting our understanding of the roles of AI-2 as a ubiquitous signal regulating overall functions within microbial communities and severely hindering large-scale functional studies of QS in the natural environment.

The development of omics technologies has facilitated research on group behaviors regulated by AI-2-mediated QS systems within complex natural environments. We addressed gaps in this research through a combination of various microbial genomic and metagenomic sequencing to investigate the mediation of microbial social communications by AI-2, as well as its signal transduction modes, which trigger a set of social behavioral responses in prokaryotes. First, we explored the key genes of the LuxS/AI-2 pathways and signal transduction modes of reference or representative genomes in prokaryotes, to demonstrate that AI-2 and its signal transduction modes are widely distributed among prokaryotes. We further used 52,515 metagenome-assembled genomes (MAGs) from 10,450 globally distributed metagenomic samples to examine the distribution of LuxS proteins and different types of receptors in global habitats. We also explored the microbial communication networks mediated by AI-2 in different ecosystem types, as well as the expression levels of *luxS* genes and various receptor genes in the mouse gut metatranscriptome. Our findings reveal the broad spectrum of social functions mediated by AI-2 in prokaryotic microbial communities.

## Results

### Prevalence of the LuxS/AI-2 pathways in prokaryotes

**Figure 1:**
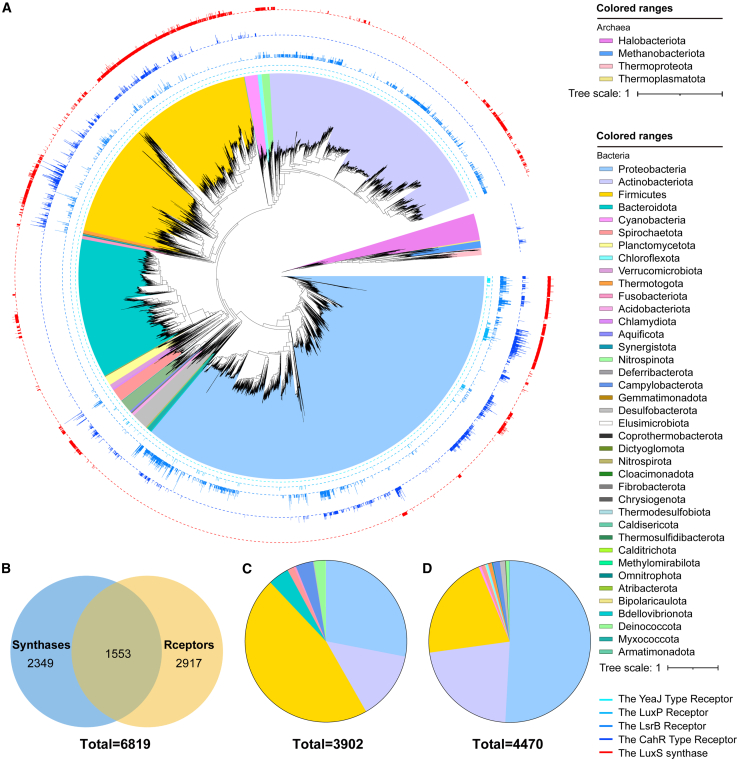
Prevalence of predicted key genes of the LuxS/AI-2 pathway in prokaryotes from RefSeq database (A) The phylogenetic tree of prokaryotic genomes from the NCBI RefSeq database. (B) Number of bacterial genomes containing LuxS proteins and AI-2 receptors. LuxP, LsrB, CahR, and YeaJ-type proteins act as AI-2 receptors. (C) Bacterial taxa coding AI-2 synthases. (D) Bacterial taxa coding AI-2 receptors. The phylogenetic tree was constructed via FastTree v2.1.10 with default parameters according to the protein sequence alignments of 120 core bacterial or 53 core archaeal genes generated by the GTDB-Tk and visualized using iTOL.

**Figure 2:**
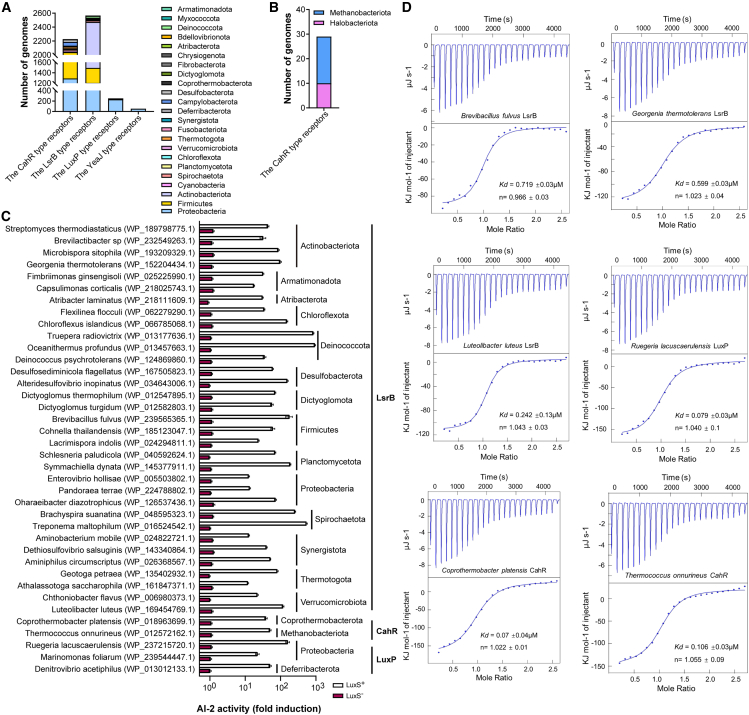
Prevalence and binding activity of predicted AI-2 receptors (A) Bacterial taxa coding four types of AI-2 receptors. (B) Archaeal taxa coding AI-2 receptors. (C) Different types of AI-2 receptors randomly selected in microbial species are capable of retaining AI-2. The binding assay was determined using *V. harveyi* MM32 bioluminescence. The data shown represented one representative of three independent experiments with similar results. (D) AI-2 binds to the different types of AI-2 receptors with high affinity. The binding affinity was determined using ITC. The data shown represented one representative of three independent experiments with similar results.

Although AI-2 acts as a QS molecule in the mammalian gastrointestinal tract,^12^^,^^15^ its occurrence and physiological role in prokaryotes remains unclear. The reference sequence (RefSeq) database at the National Center for Biotechnology Information (NCBI) is a publicly available database that contains at least one reference or representative genome for each sequenced species.^23^ We searched for key genes of the LuxS/AI-2 pathways in 14,788 bacterial and 509 archaeal genomes recorded in NCBI RefSeq with high assembly quality filtered by CheckM tool. We identified a total of 6,819 (46.11%) bacterial genomes containing AI-2-related proteins (Figures 1A and 1B), of which 3,902 (26.39%) contained LuxS proteins, these genomes belonged to 10 bacterial phyla, the most abundant of which was firmicutes, followed by proteobacteria, actinobacteria, and bacteroidetes (Figure 1C). We also identified 4,470 (30.23%) bacterial genomes containing receptors, which belonged to 23 bacterial phyla, mainly proteobacteria, actinobacteria, and firmicutes (Figure 1D). Meanwhile, it was observed that 1553 genomes contained both synthases and receptors. LsrB receptors were observed to be distributed across 15 bacterial phyla: proteobacteria, actinobacteriota, firmicutes, deinococcota, spirochaetota, chloroflexota, thermotogota, verrucomicrobiota, planctomycetota, armatimonadota, synergistota, desulfobacterota, dictyoglomota, fusobacteriota, and atribacterota (Figures 2A and 2B). This finding contradicted the traditional notion that LsrB receptors were present only in enteric bacteria and microbes from the families Clostridiaceae, Rhizobiaceae, and Bacillaceae. To determine whether the sensory domains of these receptors had the capacity to bind AI-2, we performed the *V. harveyi* MM32 reporter assay. The results showed AI-2 binding activity in 33 sensory domains selected randomly from 3,493 LsrB proteins among 14 bacterial phyla (Figure 2C), which suggested that LsrB receptors might be widespread among prokaryotes. Subsequently, a sequence alignment analysis was conducted on the LsrB-type receptor proteins that had been experimentally verified (Table S1), thereby further illustrating the plasticity of this type of receptor protein. This expansion of LsrB-type sensors represents a breakthrough in AI-2-mediated cell communication research and increases our knowledge of LsrB-type sensors. LuxP and YeaJ-type receptors were mainly found in proteobacteria (Figure 2A), in which 3 sensory domains distributed in the proteobacteria and deferribacterota randomly selected from 267 LuxP receptor proteins showed AI-2 binding activity, whereas 2 YeaJ-type receptors did not show AI-2 binding activity in firmicutes and fusobacteria (Figure 2C). A recent study demonstrated that YeaJ-type receptors are present only in Enterobacterales members,^21^ therefore, 63 YeaJ-type receptor proteins contained GAPES1 sensory domains from Enterobacterales are subjected to analyses in this study. The results showed that 2,228 bacterial genomes, mainly from the phyla proteobacteria and firmicutes, and 29 (5.70%) archaeal species from methanobacteriota and halobacteriota contained CahR-type receptors (Figures 2A and 2B), among which 2 sensory domains each from bacteria and archaea exhibited AI-2 binding activity (Figure 2C). Our binding analysis of AI-2 with randomly selected sensors by isothermal titration calorimetry (ITC) showed that AI-2 is a high-affinity ligand for these sensors (Figure 2D). Collectively, these results demonstrate the broad occurrence of AI-2 synthases *luxS* genes and sensor receptors among prokaryotes.

### Predicted AI-2 signal transduction pathways in prokaryotes

**Figure 3:**
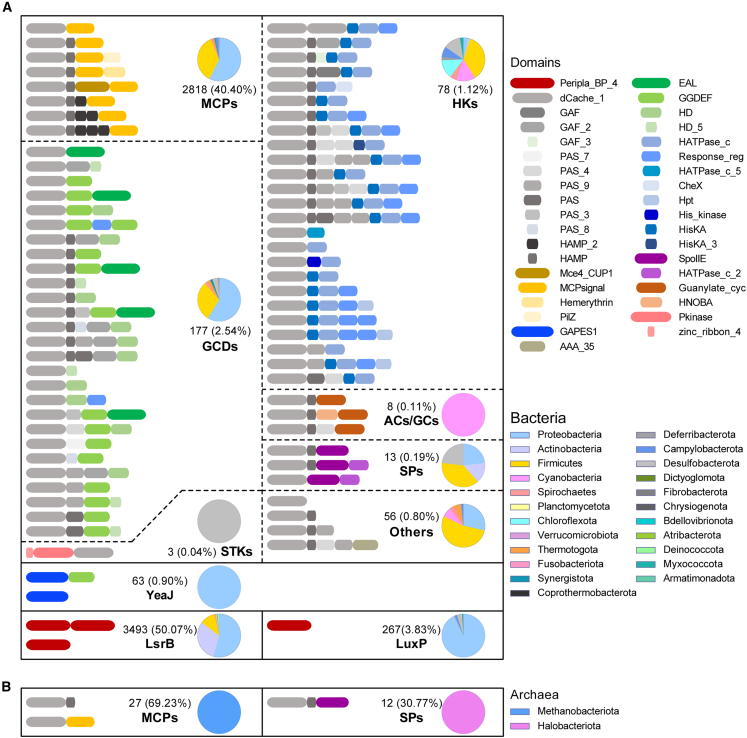
The signal transduction modes of AI-2 in prokaryotes from RefSeq database The signal transduction modes of AI-2 in bacteria (A) and archaea (B). The predicted signal transduction modes architecture of AI-2 calculated by multiple sequence alignment and hidden Markov models with the perl script pfam_scan.pl (v.1.6) against Pfam-A database (Nov 2021) with *E* value ≤10^−5^. The pie charts indicate the distribution of the signal transduction modes.

Given the ubiquity and wide distribution of AI-2, we assessed the involvement of AI-2 in signal transduction following receptor recognition, as well as its effects on the functions of complex microbial populations in prokaryotes. We found that AI-2 bound to the dCache_1 domains of CahR receptors (45.20% of receptor proteins), potentially engaging in multiple predicted AI-2 signal transduction pathways, including MCPs (e.g., Mce4_CUP1, MCPsignal, Hemerythrin, and PilZ), GCDs (e.g., EAL, GGDEF, HD, and HD_5), SPs (e.g., Spo IIE), HKs (e.g., HATPase_c, HATPase_c_5, HisKA, and HisKA_3), ACs/GCs (e.g., Guanylate_cyc and HNOBA), STKs (e.g., Pkinase), to regulate microbial phenotypes. Among these signal transduction modes, MCPs were the dominant AI-2 signal transduction proteins in bacteria, followed by GCDs, HKs, SPs, ACs/GCs, and STKs (Figure 3). Different types of signal transduction proteins perform various functions in specific microbial taxa, such as MCPs mainly in the proteobacteria, firmicutes, campylobacterota, spirochaetes, and thermotogae; GCDs in the proteobacteria and firmicutes; HKs in the firmicutes, chloroflexota, cyanobacteria, and desulfobacterota; SPs in the firmicutes, proteobacteria, and desulfobacterota; AC/GCs in the cyanobacteria; and STKs only in the desulfobacterota (Figure 3A). Only MCPs and SPs were found among the archaea, in the methanobacteriota and halobacteriota, respectively (Figure 3B). AI-2 bound to the GAPES1 domain of YeaJ-type receptors (0.90% of receptor proteins) was found to activate the GGDEF domain, which is responsible for c-di-GMP synthesis. This signal transduction mode mainly affects the functions of proteobacteria. In addition, AI-2 was observed to bind to the Peripla_BP_4 domains of LsrB and LuxP proteins for transport into the cell. LsrB and LuxP proteins were found mainly in proteobacteria (Figure 3A). These data suggest that AI-2 may exert different functions in diverse taxa through the activation of distinct receptors. To our knowledge, this study has obtained the most comprehensive information on AI-2 signal transduction modes to date.

### Transcriptomic analysis of *Streptomyces coelicolor* A3(2) an LsrB containing organism

**Figure 4:**
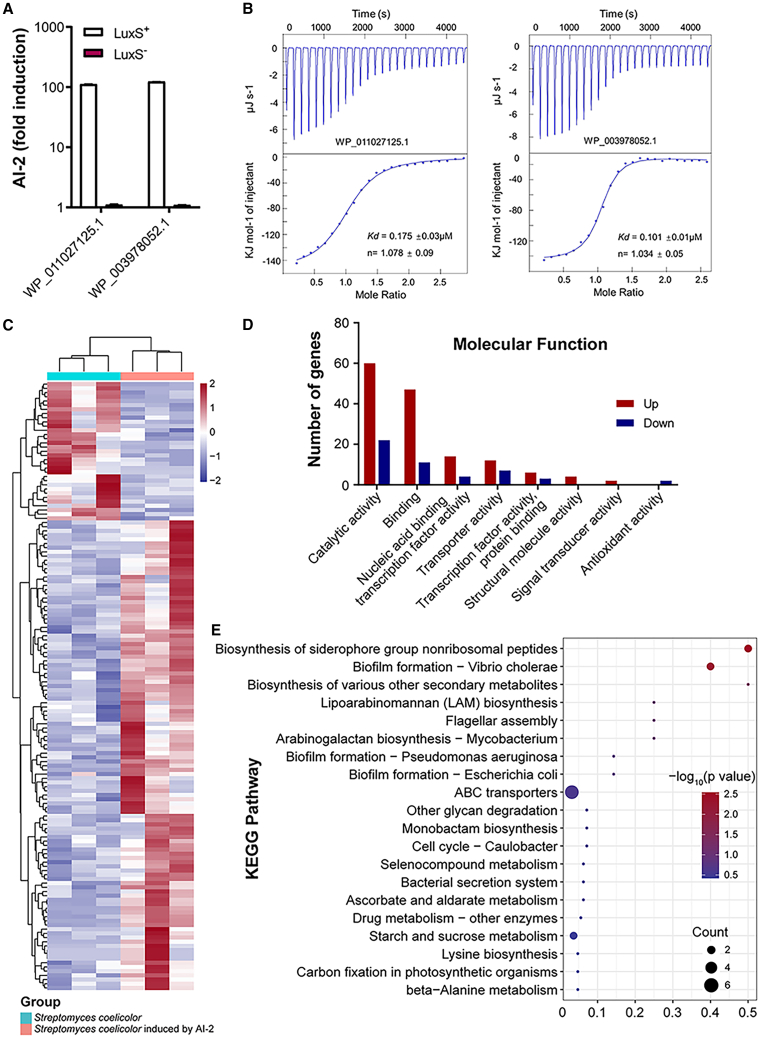
Functional profile and transcriptome analysis of *Streptomyces coelicolor* supplemented with 5 μM AI-2 or parallel controls (A) The *lsrB* genes predicted in *S. coelicolor* are capable of retaining AI-2. (B) AI-2 binds to LsrB proteins with high affinity. (C) Heatmaps of 145 DEGs. (D) The molecular function of DEGs in the *S. coelicolor* induced by AI-2. (E) Kyoto Encyclopedia of Genes and Genomes (KEGG) pathways in the *S. coelicolor* genome. All data from 3 biological replicates.

Actinomycetes are not only the dominant phylum in the prokaryotes (Figure 1A), but also have not been previously found to contain LsrB receptors. Among them, *Streptomyces* is the member with the most abundant receptors in actinomycetes. Previous studies have shown that *Streptomyces* is among the predominant members of the core microbiome of phylum actinobacteria in agroecosystems.^24^ Thus, we chose the specific *Streptomyces* for a deeper analysis. Genome re-annotation and prediction analysis identified two genes encoding LsrB in the *S. coelicolor* A3(2) genome. We investigated further the ability of these predicted LsrB receptors to bind AI-2 in the *V. harveyi* MM32 bioluminescence assay. AI-2 binding activity was observed for these receptors (Figure 4A). Furthermore, an ITC binding affinity assay showed that the LsrB receptors bound AI-2 with high affinity, with disassociation constant (Kd) values of 0.175 ± 0.03 and 0.101 ± 0.01 μM (Figure 4B). Subsequently, a sequence alignment analysis of the LsrB-type receptor proteins from *S. coelicolor* A3(2) was performed, revealing a relatively high level of site conservation, with four conserved sites identified (Table S1). These experiments demonstrated that AI-2 specifically binds to LsrB with high affinity, suggesting that among phylum actinobacteria members, LsrB receptors may function as AI-2 receptors. To understand more fully how AI-2 regulates gene expression in *S. coelicolor* A3(2) by directly engaging LsrB, we performed a transcriptomic analysis supplemented with 5 μM AI-2 or parallel controls. Notably, two clear clusters of differentially expressed genes (DEGs) were observed. A total of 145 DEGs (112 upregulated, 33 downregulated, fold change ≥2.0; *p* value ≤0.05) were detected (Figures 4C and S1). The most common molecular functions were catalytic and binding activity (Figure 4D). Several Kyoto Encyclopedia of Genes and Genomes (KEGG) pathways were significantly enriched in *S. coelicolor* A3(2) induced by AI-2, including the biosynthesis of siderophore group nonribosomal peptides and biofilm formation to regulate the metabolic and cellular processes in *S. coelicolor* (Figure 4E). These data demonstrate that AI-2 regulates gene expression in a specific *S*. *coelicolor*, and this observation indicates that it regulates physiological functions in actinobacteria members via LsrB.

### Global distribution of AI-2-related proteins

**Figure 5:**
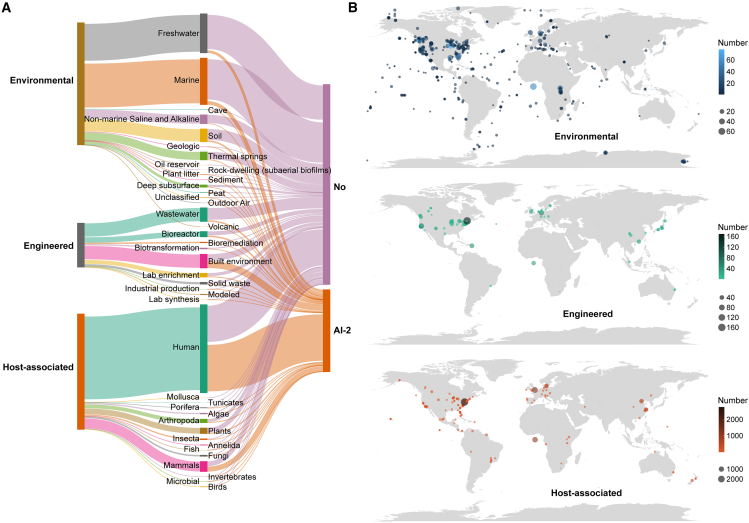
The distribution of AI-2-related genes globally across different habitats (A) Distribution of MAGs coding AI-2-related genes across different habitats. (B) Geographic distribution of samples with AI-2-related genes.

Large-scale genomic resources are critical to microbial research. We analyzed 10,450 metagenomic samples collected from habitats worldwide, including natural (22,746 MAGs), engineered (8,251 MAGs), and host-associated environments (21,518 MAGs). From this database, 52,515 MAGs were re-annotated. To determine how microbial communication based on AI-2 is distributed in different environments, we identified that there are 1,963 genomes (8.63%) in natural environments, 2,330 genomes (28.24%) in engineered environments, and 11,009 genomes (51.16%) in host-associated environments that encode AI-2 synthases and receptors (Figures 5 and S2A–S2C). Of these, 631 genomes contained LuxS proteins and 1,487 contained receptors in natural environments (Figure S2A), 1,366 contained LuxS proteins and 1,679 contained receptors in engineered environments (Figure S2B), and 10,215 contained LuxS proteins and 1,766 contained receptors in host-associated environments (Figure S2C), demonstrating the broad distribution of AI-2. These samples were divided into 26 diverse sub-habitats followed by filtration (<1‰ of total microbial genomes). We compared the abundance and composition of AI-2-related proteins among the sub-habitats and found that anthropogenically constructed habitats, mainly including urban subways, fungi, and bioreactors had higher abundance levels of AI-2 synthetases and receptors. Among the habitat categories, biotransformation, birds, humans, and mammals had higher abundance levels of AI-2 synthases LuxS proteins than receptors, whereas other categories showed the opposite trend (Figures S2D–S2F). CahR and LsrB receptors were dominant in most of the habitat categories. LuxP homologs were mainly present in the marine, freshwater, algae, and annelid environment categories, whereas YeaJ homologs mainly present in the soil, built, and human environment categories (Figure S3). These results indicate that, although AI-2-related proteins exhibit distinct abundance and composition patterns, they are widely distributed in various environments worldwide.

Next, we investigated the occurrence of AI-2 synthetases and receptors in four habitat types (oceans, soils, plant rhizospheres, and animal gastrointestinal tracts). We identified 561 (40.30%), 457 (52.41%), 554 (69.60%), and 618 (66.24%) genomes encoding AI-2-related genes in oceans, soils, plant rhizospheres, and animal gastrointestinal tracts, respectively. The abundance levels of receptors in oceans, soils, and plant rhizospheres were greater than those of synthases, and the reverse trend was observed in animal gastrointestinal tracts (Figures S4A–S4D). The largest population of CahR-type AI-2 receptors was found in four typical habitats, followed by LsrB. Ocean habitats contained the highest abundance of LuxP receptors, and soil habitats had the highest abundance of YeaJ homologs (Figure S4). The majority of synthases and CahR receptors belonged to the firmicutes and proteobacteria in oceans, soil, and plant rhizosphere environments, whereas in animal gastrointestinal tracts (Figures S5A–S5D), synthases were widely distributed among the firmicutes and actinobacteriota, and receptors were mainly distributed in the firmicutes, mainly including CahR-type receptors. Members of the proteobacteria, actinobacteria, and firmicutes contained the largest proportion of LsrB receptors. CahR receptors were observed in the methanobacteriota and halobacteriota in ocean and soil habitats (Figures S5A and S5B). Although the proportions of these receptors differed among phyla in different habitats, their relative abundance levels were consistently high. Thus, AI-2 may influence QS functions by mediating the dominant microbial groups in each habitat.

### Predicted microbial communication networks mediated by AI-2

**Figure 6:**
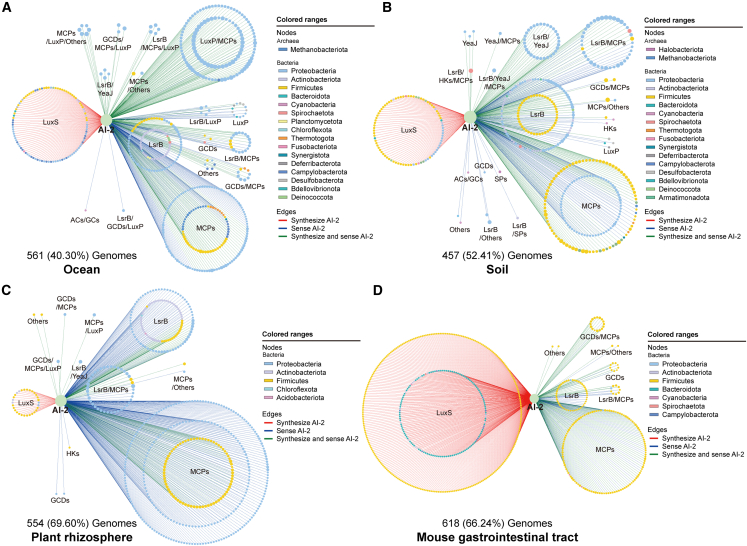
The microbial communication network mediated by AI-2 in four habitat types The microbial communication networks of the ocean (A), soil (B), plant rhizosphere (C), and mouse gastrointestinal tract (D). The colors of the nodes represent microbial phyla and AI-2 signaling molecule. Red edges indicate that the bacterial species served as “senders” produce AI-2 signaling molecules, blue edges indicate that AI-2 binds to the microbial species served as “receivers”, and green edges indicate that the bacterial species can produce and sense AI-2 signaling molecules.

To understand microbial communications more fully, we constructed predicted microbial communication networks mediated by AI-2 based on ocean, soil, plant rhizosphere, and animal gastrointestinal tract and observed 561, 457, 554, and 618 microbial species from 17, 17, 5, and 7 in these environments, respectively. In the ocean, soil, and plant rhizosphere network, the majority of these microbes belonged to proteobacteria, followed by firmicutes, and actinobacteria, whereas in the animal gastrointestinal tract network, phylum firmicutes showed the greatest abundance, followed by bacteroidetes. In marine and soil networks, approximately 18% of bacterial strains produce AI-2 as a communication language, >40% of bacterial species both produce and respond to AI-2, and >35% of microbes have the receptors but do not produce the signals due to lacking the *luxS* genes, which indicates that they may require external source of AI-2 to respond to regulate behaviors by QS. In the plant rhizosphere, only 30 (of 554, 5.42%) of bacterial species produce AI-2, and 70.76% (392 of 554) sense AI-2, whereas in gastrointestinal tract, 67.15% (415 of 618) produce AI-2 and 11.00% (68 of 618) have predicted receptors and thus have the potential to respond to AI-2. More than 20% of bacteria in the plant rhizosphere and animal gastrointestinal tract, both produce and sense AI-2 (Figures 6A–6D). These data show that communications were universal in different habitats, but with distinct modes.

**Figure 7:**
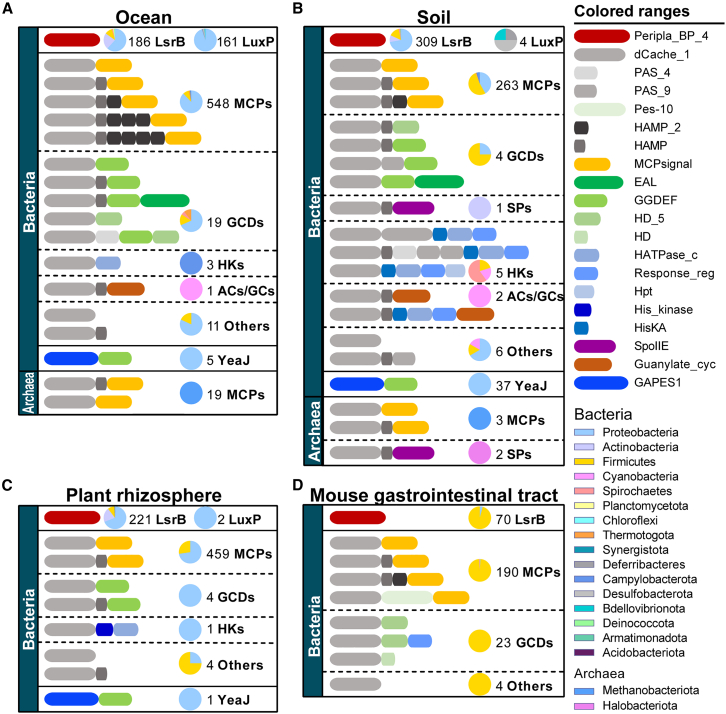
The signal transduction modes of AI-2 in four habitat types The signal transduction modes of AI-2 in ocean (A), soil (B), plant rhizosphere (C) and mouse gastrointestinal tract (D). The pie charts show the distribution of the signal transduction modes.

Moreover, we found that AI-2 performed their functions by multiple type receptor proteins to regulate social behaviors. Multiple types of AI-2 sensors were observed including Peripla_BP_4 domain of LsrB and LuxP proteins, dCache_1 domains of CahR-type receptors and the GAPES1 domain of YeaJ-type receptors. The LsrB and LuxP proteins contain only the sensor domain, CahR protein contains a variety of known functional domains or kinase domains, such as MCP, EAL, GGDEF, HD, HD_5, Spo IIE, and YeaJ hosts GGDEF functional domains following the sensor domain, which is responsible for c-di-GMP synthesis. Marine, soil, and plant rhizosphere were prescribed more classes of functional domains than animal gastrointestinal tract. MCPs were dominant receptor proteins across different habitants, GCDs were more in marine and animal gastrointestinal tract (Figure 7). The diversity of AI-2 sensors and difference of distribution suggests that the complexity of cell-cell communication, and the diversity of QS functions among microbiota mediated by AI-2. To sum up, the microbial communication networks contained a broader degree of functional diversity mediated by AI-2 than previously appreciated. To evaluate the expression of *luxS* genes and receptor genes in the natural bacterial community, we randomly selected mouse gut microbial metatranscriptomic datasets and calculated the expression levels of *luxS* genes, CahR-type receptor genes and *lsrB* genes in 195 (of 550, 35.45%), 43 (of 157, 27.39%), and 34 (of 55, 61.82%) genomes, respectively, within these datasets (Figure S6A). firmicutes and bacteroidetes were the predominant phyla in terms of the expression of AI-2-related genes (Figure S6B). Among these, *luxS* genes were expressed in the firmicutes and bacteroidetes, whereas most receptors expressed in the firmicutes (Figures S6A and S6B). On average, more *luxS* genes were expressed among members of the Lactobacillales, particularly *Ligilactobacillus murinus* (GCA_910578875.1), followed by members of the Anaerotignaceae (GCA_910588385.1). The highest number of receptor genes were expressed in members of the Lachnospiraceae, such as *Schaedlerella* sp. (GCA_910587215.1 and GCA_910589385.1), which expressed *lsrB* genes and *Butyribacter* sp., which expressed CahR-type receptor genes. The expression of synthetase and receptor genes originally detected within members of the Lachnospiraceae (GCA_910579345.1 and GCA_910585295.1) was also observed (Figure S6C). These results validated our analytical results that AI-2 related genes are expressed in different habitats.

## Discussion

The social behaviors of microbes rely on complex biological processes that require cell-cell communication. Multiple studies have revealed that AI-2 mediates the cell communications and enables them to modify their diverse phenotypes in many species such as *V. cholerae*,^13^ *E. coli*,^14^ and *P. aeruginosa*,^25^ and contribute to the rumen microbe functions^15^ and gut microbial population balance.^12^ Given the widespread and important roles of AI-2 in the microbial world, it is crucial to elucidate the mediation of events related to AI-2. Microbial communication relies on the transmission of information (signaling molecules) from “senders” to “receivers”, where “receivers” are equipped with sensory abilities specialized to allow those signals to influence social behaviors of the microbial consortium. The *luxS* gene acts as a “sender” and is responsible for synthesizing AI-2.^16^ LuxP, LsrB,^25^ CahR,^20^ and YeaJ-type AI-2 receptors^21^ act as “receivers”, and are responsible for transmitting AI-2 signals into cells.^16^^,^^17^ Many bacteria with LuxS but without any of the known receptors were observed, indicating that there may be some bacteria with unknown receptors that still need to be discovered and require further research in the future. Meanwhile, it was observed that several types of receptors might be caused by different environments, such as some bacteria having LsrB and others having LuxP can be related with the fact that bacteria are adapting to sense the different forms of AI-2 (borated and unborated) present in the different environments the bacteria live in. The different forms of the signal are related to the chemical composition of the environment (e.g., boron or no boron, or different concentrations of boron), thus bacteria may have evolved to detect different forms of AI-2 depending on the chemical properties of the environment they live in. Prior knowledge helps us to understand more fully how AI-2-related proteins influence microbial functions in monospecies cultures and may affect social behaviors. In this study, we investigated intact LuxS/AI-2 signaling pathways, in which “senders” are responsible for the biosynthesis of the signal and specific “receivers” perceive and transmit the signal. We also investigated the mechanism underlying AI-2 signal transduction after AI-2 sensor recognition and its influence on the functions of complex microbial populations. Our findings constitute a substantial contribution to the current knowledge on LuxS/AI-2, deepening our understanding of the functions of AI-2 during social processes in a genomics context, and providing clues for future studies to characterize the related functional mechanisms.

Several studies have reported phenotypes mediated by AI-2 in a wide variety of bacteria in monospecies cultures, and others have investigated the mechanisms of microbial consortium social behaviors under the control of AI-2 on a global scale. We analyzed 15,297 prokaryotic genomes from the RefSeq database and found that approximately 46% of these genomes contained genes encoding AI-2-related proteins, approximately 30% of which contain AI-2 receptor-encoding genes, including the LuxP, LsrB, CahR, and YeaJ-type AI-2 receptors. This study demonstrated for the first time that LsrB receptors are found in 17.35% of bacterial reference genomes and distributed across 15 bacterial phyla, which is inconsistent with findings from previous studies. Concurrently, we conducted luminescence and ITC experiments for verification (Figures 2C, 2D, 4A, and 4B). Subsequent sequence analysis indicated that, although the conservation of certain proteins compared to the LsrB receptor in *Salmonella* has diminished, these proteins are still capable of binding to AI-2 (Table S1). This finding aligns with previous study results indicating the plasticity of LsrB-type receptor proteins. This will help to more accurately and comprehensively identify potential LsrB-type receptors, thus promoting future research. LsrB receptors were initially reported to bind AI-2 in *S.* typhimurium.^26^ Indeed, it is also present in other enteric bacteria and members of the Clostridiaceae, Rhizobiaceae and Bacillaceae families.^17^^,^^19^ They belonged to the proteobacteria and firmicutes. *Streptomyces* is the member with the most abundant receptors in actinomycetes, which have not been previously found to contain LsrB receptors. Hence, given that the well-established AI-2 receptor LsrB plays a role in the internalization of AI-2 in only some species,^27^ we investigated the mechanism by which AI-2 regulates metabolism and cellular processes in a specific *Streptomyces* containing an LsrB receptor. Our findings demonstrated the likely involvement of AI-2 associated with the biosynthesis of siderophore group nonribosomal peptides and biofilm formation via LsrB in *Streptomyces* species. Future studies of the multiple biological functions of different receptors will extend our knowledge of the functions and molecular mechanisms of AI-2 in more complex ecosystems.

In addition to the importance of AI-2 in the prokaryotic reference genome, AI-2 has also been demonstrated to play roles in microbial populations.^12^^,^^15^ However, studying the complex ecological functions of signaling molecules within distinct microenvironments is particularly challenging due to the high microscopic complexity within ecosystems and technological limitations associated with its representation. We re-annotated 52,515 MAGs from 10,450 metagenomic samples to identify LuxS proteins and receptors, which allowed us to gain an unprecedented view of the distribution of LuxS proteins and receptors across different habitats. A total of 15,302 MAGs were found to contain LuxS proteins and receptors from various habitats, indicating that communications mediated by AI-2 appear to be widely distributed in diverse environments worldwide. The ability to determine the spatial distribution of LuxS proteins and receptors across different habitats will transform our understanding of their influence on microbial functions, including social functions, based on the LuxS/AI-2 pathways. In addition to anthropogenically disturbed microenvironments such as biotransformed substrates, vastly more “senders” than “receivers” were found among certain habitat categories, i.e., birds, humans, and mammals; it is possible that cross-kingdom communication occurs between mammalian epithelial cells and their bacteria, mediated by AI-2 mimics.^28^ This increase in signaling molecules suggests the potential for competitive binding to target receptors within a certain range. Furthermore, LsrB and CahR-type receptors were by far the most abundant across diverse habitats, such as ocean, soil, plant rhizosphere, and animal gastrointestinal tract habitats, which is inconsistent with our previous finding that CahR-type receptors are more numerous than LsrB and LuxP among rumen bacteria.^15^ This phenomenon may be explained by a recent study, which demonstrated that interactions among microbes within a consortium and the physiochemical characteristics of microenvironments may ultimately shape differential community structures, leading to the construction of a varied microbial communication network.^29^^,^^30^^,^^31^^,^^32^ Further research is needed to understand this phenomenon. Almost all animals make decisions and adopt behaviors depending on multiple sensory systems^33^; we explored the biological behaviors among microbes across a prokaryotic database and global natural habitats from the perspective of AI-2 as a sensor. We further investigated how the signal transduction modes of AI-2 after AI-2 sensor activation affect the functions of complex microbial populations in prokaryotes. Our findings open new avenues for discovering interrelationships among microbes that are important for complex natural ecosystems. Future studies should investigate further the microbial regulation of these complex relationships and their influence on social behavior, to obtain a comprehensive understanding of the precise mechanisms driving communication within microbial consortia in response to complex environmental changes.

### Limitations of the study

To the best of our knowledge, it is a comprehensive analysis which evaluates the cell-to-cell communication mediated via the AI-2 signal. However, we acknowledge that this study has several limitations. Firstly, although we used several datasets from public databases, these might not be fully representative of real-world data as additional microbial genomes become available. Second, it is important to note that considerable further experimental work is required to validate computational predictions generated by current research, in particular the quantification of microbial interactions across habitat types in other settings. Additionally, existing research is largely focused on the monospecies cultures of microbial species, the impact of QS signals on complex microbial communities within microecosystems remains unclear. Our current knowledge of QS signals and its function is mainly based on multi-omic approaches. With the development of more effective technologies and the conduct of more high- quality studies, future studies are warranted to get insight into more detailed mechanisms for cell-cell communication within the real consortium.

## Resource availability

### Lead contact

Further information and requests for resources and reagents should be directed to and will be fulfilled by the corresponding author Xihui Shen (xihuishen@nwsuaf.edu.cn).

### Materials availability

This study did not generate new unique reagents. The materials generated in this study are available from the lead contact upon request with a completed material transfer agreement.

### Data and code availability

• A total of 14946 bacterial and 510 archaeal reference or representative genomes were downloaded from the NCBI RefSeq database http://www.ncbi.nlm.nih.gov/refseq/ (May. 2022). A total of 52,515 microbial metagenome-assembled genomes (MAGs) from 10,450 globally distributed metagenomic samples were downloaded from https://genome.jgi.doe.gov/GEMs. The 1403 marine^34^ and 928 soil^35^ microbial genomes were employed from publicly available datasets https://www.ncbi.nlm.nih.gov/sites/batchentrez. The 933 microbial genomes from mouse gastrointestinal tract^36^ are available at NCBI BioProject https://www.ncbi.nlm.nih.gov/bioproject/PRJEB45234/. The 1102 rhizosphere genomes are available at the EBI ENA https://www.ebi.ac.uk/ena/browser/text-search?query=rhizosphere (Jul. 2022). The mouse gut microbial *meta*-transcriptomic datasets were obtained from the EBI ENA with accession number E-MTAB-3562 and E-MTAB-4082. All other data reported in this paper will be shared by the lead contact upon request.
• The computational scripts in this work are available on GitHub (https://github.com/Liulab-CAAS/Autoinducer-2).
• Any additional information required to reanalyze the data reported in this paper is available from the lead contact upon request.

## Acknowledgments

This work was supported by the grant of the National Natural Science Foundation of China (31725003 and 32330004 to X.S.), the Shaanxi Fundamental Science Research Project for Chemistry and Biology (grant no. 22JHZ008 to X.S.), the Natural Science Foundation of Inner Mongolia Autonomous Region (2024QN03028 to X.L.), and the Agricultural Science and Technology Innovation Program of CAAS (CAAS-ASTIP-IGR-2023 to X.L.). We thank the Life Science Research Core Services (Luqi Li), Northwest A&F University for technical support.

## Author contributions

X.L., L. Zhang., Xiaoqing Zhang, and X.S. conceived the ideas and designed the study. Unless otherwise specified, X.L. and Xiaoxue Zhang performed all of the computational analyses. Z.W., C.L., and L.Z. performed protein expression and purification, and AI-2 binding assay experiments. M.Y. performed the transcriptomic analysis and experiments of *S*. *coelicolor* A3(2) regulated by AI-2. Z.W. and Xiaoxue Zhang performed the molecular docking simulation and conserved site analysis. X.L., Z.W., and S.L. collected the data. X.L., Xiaoqing Zhang, and X.S. wrote and revised the manuscript. All authors read and approved the final manuscript.

## Declaration of interests

The authors declare no competing interests.

## STAR★Methods

### Key resources table

**Table:** 

REAGENT or RESOURCE	SOURCE	IDENTIFIER
**Bacterial Strains**		

*Streptomyces coelicolor* A3(2)	N/A	N/A

**Critical Commercial Assays**		

IPTG	Solarbio	Cat# I8070; CAS: 367-93-1
NaH_2_PO_4_	Sigma-Aldrich	Cat# S3139; CAS: 7558-80-7
NaCl	Sigma-Aldrich	Cat#S3014; CAS: 7647-14-5
DTT(dithiothreitol)	Solarbio	Cat# D8220; CAS: 3483-12-3
Sephadex-G25	Sigma-Aldrich	Cat# S5772; CAS: 9041-35-4
Microtiter plates	Corning	Cat#3603
Tris	Solarbio	Cat# T8060; CAS: 77-86-1
Glycerol	Sigma-Aldrich	Cat# G5516; CAS: 56-81-5
DPD/AI-2	IsoReag	Cat# IR-29271; CAS: 142937-55-1
MgSO_4_·7H_2_O	Sigma-Aldrich	Cat# M2773; CAS: 10034-99-8
Casein acids Hydrolysate	Solarbio	Cat# C8221; CAS: 65072-00-6
K_3_PO_4_	Sigma-Aldrich	Cat# RDD019; CAS: 7778-53-2
L(+)-Arginine	Sigma-Aldrich	Cat# A8094; CAS: 74-79-3
Ribofavin	Solarbio	Cat# V8060; CAS: 83-88-5
VitaminB1	Solarbio	Cat# V8020; CAS: 67-03-8

**Deposited Data**		

Reference or representative genomes	RefSeq	http://www.ncbi.nlm.nih.gov/refseq/
A genomic catalog of Earth’s microbiomes	JGI Genome portal	https://genome.jgi.doe.gov/GEMs
Marine microbial MAGs	Marine Metagenomics	https://mmp2.sfb.uit.no/marref/
Soil microbial MAGs	Choi et al.^35^	https://www.ncbi.nlm.nih.gov/
Rhizosphere microbial MAGs	European Nucleotide Archive	https://www.ebi.ac.uk/ena/browser/text-search?query=rhizosphere
Mouse gastrointestinal tract microbial MAGs	Beresford-Jones et al.^36^	[BioProject]: [PRJEB45234]
Mouse gut microbial meta-transcriptomic data	European Nucleotide Archive	E-MTAB-3562 and E-MTAB-4082
LuxS profile HMM	Pfam	http://pfam.xfam.org/family/PF02664
Periplasmic binding proteins profile HMM	Pfam	http://pfam.xfam.org/family/PF13407
dCache_1 domain profile HMM	Pfam	http://pfam.xfam.org/family/PF02743
GAPES1 domain profile HMM	Pfam	https://pfam.xfam.org/family/PF17155.4

**Software and Algorithms**		

CheckM v1.0.18	Parks et al.^37^	https://github.com/Ecogenomics/CheckM
HMMER v.3.3	Sun and Buhler^38^	http://hmmer.org/
Prokka v1.14.6	Seemann^39^	https://github.com/tseemann/prokka
GTDB-Tk v2.1.0	Chaumeil et al.^40^	https://github.com/Ecogenomics/GTDBTk
FastTree v2.1.10	Price et al.^41^	http://www.microbesonline.org/fasttree/
iTOL v3	Letunic and Bork^42^	https://itol.embl.de/
Trimmomatic v0.39	Bolger et al.^43^	https://github.com/usadellab/Trimmomatic/releases
fastqc v0.11.8	Andrews et al.^44^	https://github.com/s-andrews/FastQC
multiqc v1.5	Ewels et al.^45^	https://github.com/MultiQC/MultiQC/releases
STAR v2.7.10a	Dobin et al.^46^	https://github.com/alexdobin/STAR/releases
RSEM v1.3	Li and Dewey^47^	https://github.com/deweylab/RSEM
PfamScan v.1.6	Mistry et al., 2007^51^	https://github.com/aziele/pfam_scan
GraphPad Prism 8.0.2	GraphPad Software	http://www.graphpad.com/

### Experimental model and subject details

#### Microbe strains

The bacterial strain used in this study is *Streptomyces coelicolor* A3(2), which was cultivated with Tryptic Soy Broth medium at 28°C.

### Method details

#### Data collection

This study used several datasets for comprehensively evaluating the AI-2-mediated microbial communications in prokaryotes. We downloaded 14946 bacterial and 510 archaeal reference or representative genomes retrieved from the RefSeq database on May 2022 (http://www.ncbi.nlm.nih.gov/refseq/)^23^ and obtained 52,515 metagenome-assembled genomes (MAGs) from 10,450 globally distributed metagenomic samples for investigating these communications across different habitats (https://genome.jgi.doe.gov/GEMs).^48^ For more accurately examining the communication network in important habitats, four datasets, including marine,^34^ soil,^35^ plant rhizosphere, and mouse gastrointestinal tract,^36^ were employed from publicly available datasets. To validate the performance of our results, the metatranscriptome of the mouse gut^49^ were also collected to assess the expression of these genes in real microbiome projects.

#### The genomic data processing and AI-2-related proteins prospecting

The completeness and contamination of each microbial genome was evaluated via the lineage_wf workflow with default parameters of CheckM v1.0.18.^37^ High-quality bins defined as completeness ≥80% and contamination ≤10 were retained. We explored the predicted target proteins based on hmmsearch^38^ and semantic approaches.^39^ To identify S-Ribosylhomocysteinase (LuxS) family proteins, periplasmic binding proteins homologous,the Gammaproteobacterial periplasmic sensor (GAPES1) domain-containing AI-2 receptors, and the dCache_1 domain-containing proteins, the profile HMMs, including LuxS (http://pfam.xfam.org/family/PF02664), periplasmic binding proteins profile HMM (http://pfam.xfam.org/family/PF13407), the GAPES1 domain (https://pfam.xfam.org/family/PF17155), and the dCache_1 domain (http://pfam.xfam.org/family/PF02743) were used to query all protein-coding sequences in each genomic database by hmmsearch in HMMER v.3.3 (--cut_ga parameter; Nov 2019; http://hmmer.org/), and discarded families with E-value ≤10^−5^. Proteins containing LuxS and GAPES1 domains homologous were defined as AI-2 synthases and YeaJ receptors, respectively. Conserved domain amino acid sequences of the predicted dCache_1 domain were aligned to PctA-LBD of *P. aeruginosa* via hmmsearch with default parameters. The CahR receptors contained five conserved amino acid residues (R126, W128, Y144, D146, and D173) same as these in PctA-LBD.^15^^,^^20^ In addition, each genome after filtering was reannotated based on Prokka v1.14.6 with the default settings.^39^ Both LsrB and LuxP AI-2 receptors were periplasmic binding proteins homologous, thus, they contained Peripla_BP_4 domain. To ensure accuracy, the following search terms were prospected: S-ribosylhomocysteine, diguanylate cyclase, LsrB, and LuxP.

#### The construction of phylogenetic trees based on microbial genomes

The microbial genomes (bacterial and archaeal genomes) obtained from the RefSeq database, marine, soil, plant rhizosphere, and mouse gastrointestinal tract were taxonomically classified according to the GTDB-Tk reference data r207 via GTDB-Tk v2.1.0 with the default settings.^40^ The phylogenetic tree was constructed according to the protein sequence alignments of 120 core bacterial genes or 53 core archaeal genes generated by the GTDB-Tk.^40^ Finally, we built phylogenetic trees of 14788, 509, 1392, 872, 795 and 933 microbial genomes from bacterial reference genome, archaeal reference genome, marine, soil, plant rhizosphere, and mouse gastrointestinal tract genomes via FastTree v2.1.10 with default parameters^41^ and visualized using iTOL.^42^

#### Microbial communication network mediated by AI-2

The microbial species related to AI-2 signaling molecule were classified and integrated into Excel spreadsheets to construct a microbial communication network based on AI-2, which was visualized with the Cytoscape program software.^15^^,^^50^ The major microbial taxa, signal molecule, protein types and possible link were labeled in these networks.

#### The signal transduction modes architecture of AI-2

The identified LsrB, LuxP, CahR and YeaJ-type AI-2 receptors proteins further refined by multiple sequence alignment and hidden Markov models with the perl script pfam_scan.pl (v.1.6)^51^ against Pfam-A database (Nov 2021) with E-value ≤10^−5^ to predict the signal transduction modes architecture of AI-2.

#### The gene expression within mouse gut microbial metatranscriptomes

The mouse gut microbial metatranscriptomic datasets were obtained from the EBI ENA with accession number E-MTAB-3562 and E-MTAB-4082,^49^ which contained 33 different samples. The raw data is trimmed to remove both low-quality reads and adapters of each read using Trimmomatic v0.39^43^ with parameters of SLIDINGWINDOW:5:20 after quality determined with fastqc v0.11.8^44^ and multiqc v1.5,^45^ then aligned to the mouse gastrointestinal tract gut microbial reference genomes using STAR v2.7.10a^46^ with default settings. The gene expression was calculated using RSEM v1.3^47^ with rsem-calculate-expression. Finally, The TPM values of genes of interest were extracted.

#### *In vitro* AI-2 binding assays

*E. coli* strain BL21 (DE3) and its *luxS*-deficient mutant, both harboring pET-28a derivatives with a DNA fragment encoding the LBDs of transmembrane proteins, were initially cultured at 37°C to an OD600 of 0.6. Isopropyl β-D-1-thiogalactopyranoside (IPTG) was then added to a final concentration of 0.25 mM, and the cells were grown for an additional 7 h at 16°C. Following purification with Ni-nitrilotriacetic acid (Ni2+-NTA) His-bind resin (Novagen, Madison, WI) according to the manufacturer’s instructions, the His6-tagged proteins were swapped into a buffer containing 50 mM NaH2PO4 (pH 8.0), 300 mM NaCl, and 1 mM dithiothreitol using Sephadex-G25 agarose. The purified proteins were concentrated to approximately 10 mg/mL and denatured by heating at 70°C for 10 min to release any bound ligands. The denatured proteins were pelleted, and the supernatants were subsequently tested for the presence or absence of AI-2 using luminescence assays.^20^ For this assay, an overnight culture of *Vibrio harveyi* MM32 grown in AB medium were diluted 1:5000 into fresh AB medium, and 90 μL aliquots of the diluted cells were added to 96-well microtiter plates (Corning cat# 3603). Subsequently, 10 μL aliquots of the supernatants from denatured proteins or a buffer control were added to the wells and the microtiter plates were incubated at 30°C for 8 h with shaking at 170 rpm. Bioluminescence (counts per second) was measured using microplate reader Victor X3 (PerkinElmer, Waltham, MA, USA) and AI-2 activity is reported as fold induction relative to the light production induced by the buffer control.

#### Isothermal titration calorimetry (ITC)

The interactions between proteins and DPD were assessed using isothermal titration calorimetry (ITC) at 25°C with a NANO-ITC 2G microcalorimeter (TA Instruments, USA).^52^ Briefly, the proteins and chemicals were prepared in ITC buffer (50 mM Tris, 150 mM NaCl, 10% [v/v] glycerol, pH 8.0), and DPD (IsoReag) was dissolved in the same buffer. To detect interactions between proteins and DPD, 400 μM DPD was loaded into the syringe compartment (250 μL), while the protein solution (30 μM) was placed into the microcalorimetric cell (950 μL). The stirring speed was set to 300 rpm and 25 injections were performed for each experiment. Once a stable baseline was achieved, the DPD titration was carried out with 25 injections of 10 μL into the protein solution until the protein sample was saturated with DPD. Three independent experiments were performed for each sample. Blank titration, where the DPD solution was injected into the dialysis buffer, was performed to correct for the dilution heat of the DPD solution. ITC data were analyzed and fitted to a one-site independent binding model using NanoAnalyze software version 3.4 (provided by the manufacturer), with the dilution heat subtracted from the experimental titration prior to analysis.^53^

#### Transcriptomic analysis of *Streptomyces coelicolor* A3(2)

To prepare samples for transcriptome sequencing (RNA-Seq) analysis, the *S. coelicolor* A3(2) strain was grown in 100% TSB liquid media 2 days at 28°C with shaking at 220 rpm, followed by culture in fresh YEME liquid media (1% final inoculation volume) until the mid-exponential growth phase. *S. coelicolor* A3(2) were further cultivated for 2 h after supplementation with 5 μM AI-2 or parallel controls. Subsequently, the cells were harvested by centrifugation at 6,000 rpm for 3 min at 4°C for total RNA extraction using a TRIzol-based method (Life Technologies, CA, USA) following the manufacturer’s instructions. RNA quality control was assessed using 1% agarose gel electrophoresis and a Thermo Scientific NanoDrop 2000 spectrophotometer. The RNA samples were then used for cDNA synthesis. Firstly, using the illumina MRZB12424 Ribo-Zero rRNA Removal Kit (Bacteria) (Illumina, San Diego, CA, USA) to remove the rRNA from total RNA. Then, first-strand and second-strand cDNA were synthesized using ProtoScript II Reverse Transcriptase (New England BioLabs, Ipswich, MA, USA) and the NEBNext Second Strand Synthesis Reaction Buffer and dATP, dGTP, dCTP, dUTP mix (New England BioLabs, Ipswich, MA, USA), respectively. The resulting cDNA was purified using Agencourt AMPure XP beads (Beckman Coulter, Brea, CA) and end repaired with NEBNext End Repair Reaction Buffer and Enzyme Mix (New England BioLabs, Ipswich, MA, USA). Sequencing adapters were ligated using NEBNext Adaptor for Illumina (New England BioLabs, Ipswich, MA, USA) according to standard protocols. Following that, the second-strand cDNA was degraded using the USER enzyme mix (New England BioLabs, Ipswich, MA, USA) and the product was purified using Agencourt AMPure XP beads (Beckman Coulter, Brea, CA). Finally, the index-coded samples were clustered on a cBot Cluster Generation System using the NEBNext Q5 Hot Start HiFi PCR Master Mix (New England Biolabs, Ipswich, MA, USA). After cluster generation, sequencing was performed using the Illumina NovaSeq 6000 platform with paired-end 150 base reads.^54^^,^^55^

#### Molecular docking analysis

The protein structures were predicted by AlphaFold3 and subsequently optimized with the Protein Preparation Wizard^56^ implementation in the Schrödinger suite (Schrödinger Release 2018-4: Schrödinger, LLC, New York, NY, 2018). The DPD structure was obtained directly from the PubChem database. Flexible torsions of proteins and molecules were assigned using the Autotors utility within AutoDock4, hydrogens were then added and the Gasteiger charges were calculated. Docking simulations were performed using AutoDock4 Vina 1.1.241 and the optimal binding mode was selected based on the lowest docking energy.

### Quantification and statistical analysis

Statistical analysis was performed using GraphPad Prism 8.0.2 (GraphPad Software, San Diego, CA). Data were assessed for normal distribution and plotted in the figures as mean ± SEM. No samples were excluded from the analyses. The ITC data of the binding constant (*K*_*d*_) and binding stoichiometry (N) were calculated using Nano Analyze software version 3.4, provided by the manufacturer and fitted with a one-site independent binding model.

## Supplemental information

Document S1. Figures S1–S6 and Table 1

Data S1. Raw Data
