# Supplementary material for: A global perspective on autoinducer-2-mediated cell communication in prokaryotes
Source: iScience. 2025 Jun 13;28(7):112908. doi: 10.1016/j.isci.2025.112908 (PMC12268683; doi:10.1016/j.isci.2025.112908)
Supplement: Document S1. Figures S1–S6 and Table 1 [file mmc1.pdf]

iScience, Volume *28*

Supplemental information

A global perspective on autoinducer-2-mediated cell communication in prokaryotes

Xiaozhen Liu, Zhiyan Wei, Mingming Yang, Xiaoxue Zhang, Zhuo Wang, Shuyu Li, Changfu Li, Lingfang Zhu, Lei Zhang, Xiaoqing Zhang, and Xihui Shen

Supplemental Figures

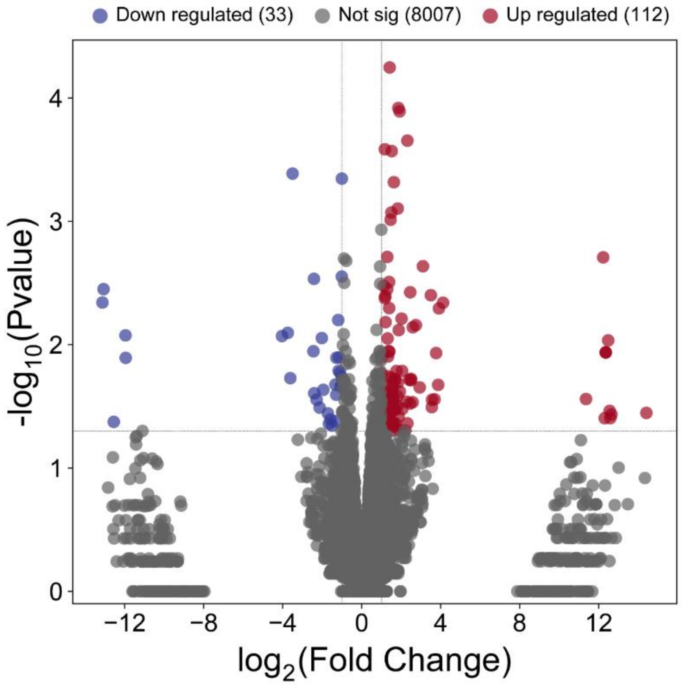

Figure S1. Transcriptome analysis of genes in the *Streptomyces coelicolor* induced by AI-2. Volcano plot of all transcripts identified in the *Streptomyces coelicolor* A3 induced by AI-2. The red gene is upregulated and the blue gene is downregulated.

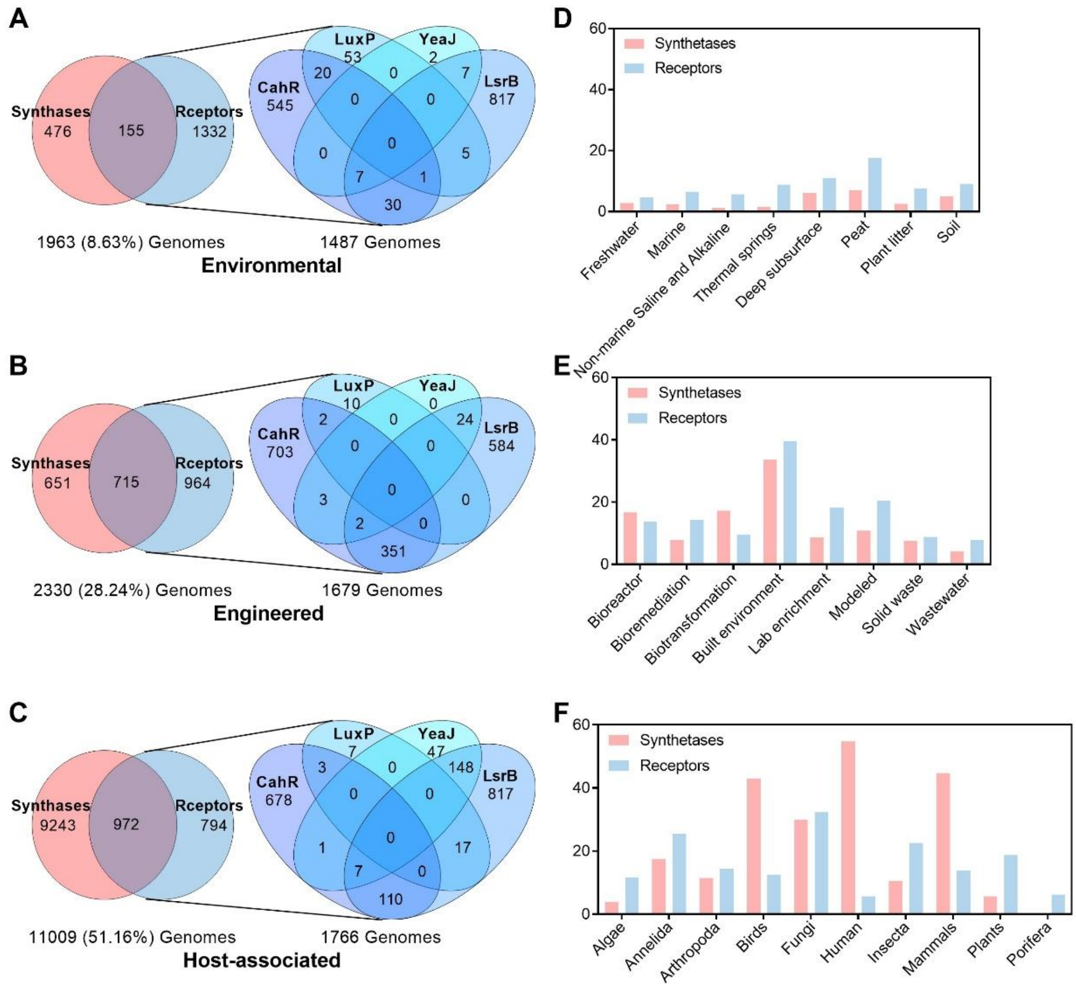

Figure S2. The abundance of AI-2-related proteins globally across different habitats. Abundance of AI-2 synthases and receptors in the natural environments **A**, engineered environments **B**, and host-associated environments **C**, respectively**.** The abundance and distribution of AI-2 synthases and receptors in each sub-habitat of the natural environments **D**, engineered environments **E** and host-associated environments **F**, respectively. These samples were divided into 26 diverse sub-habitats followed by filtration (<1‰ of total microbial genomes).

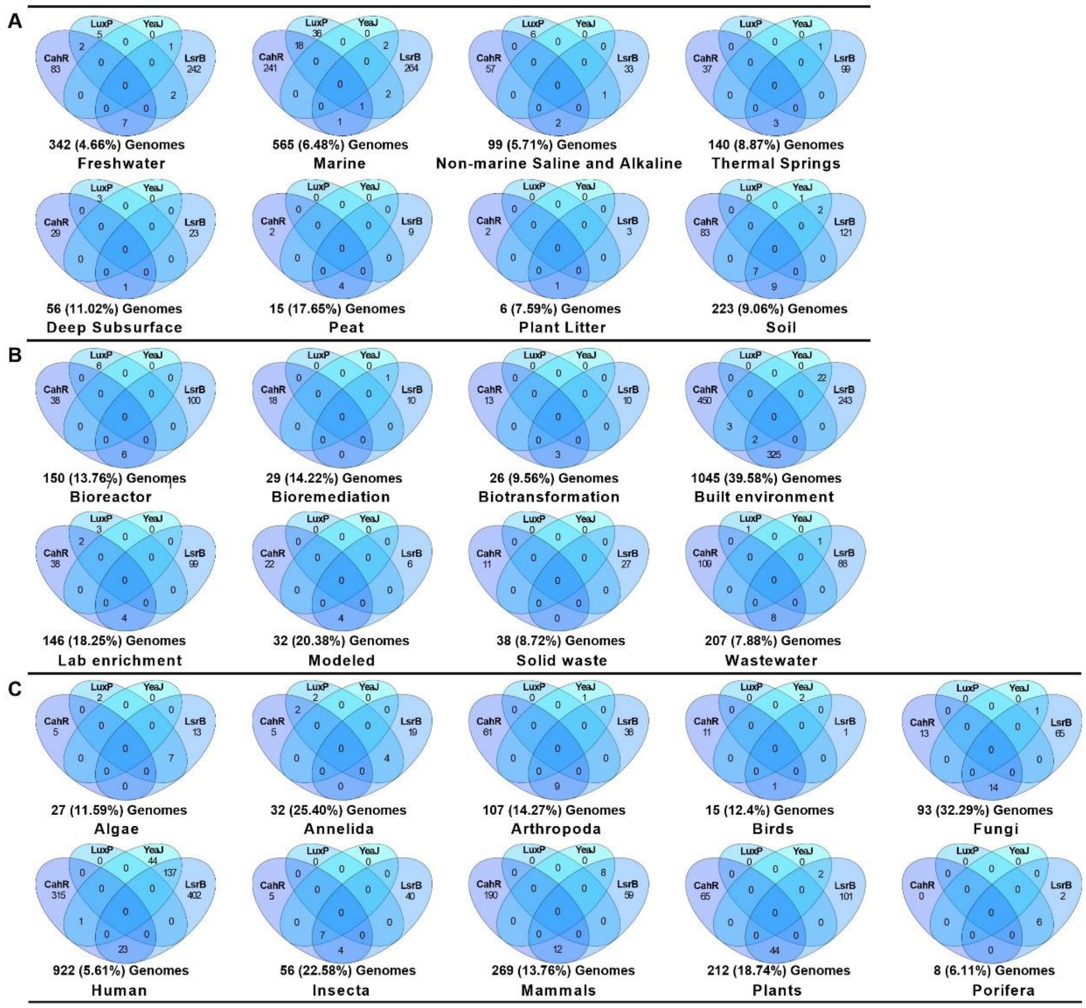

Figure S3. The abundance of AI-2 receptors globally in each sub-habitat of different habitats. Abundance of AI-2 receptors in each sub-habitat of the natural environments **A**, engineered environments **B**, and host-associated environments **C**, respectively.

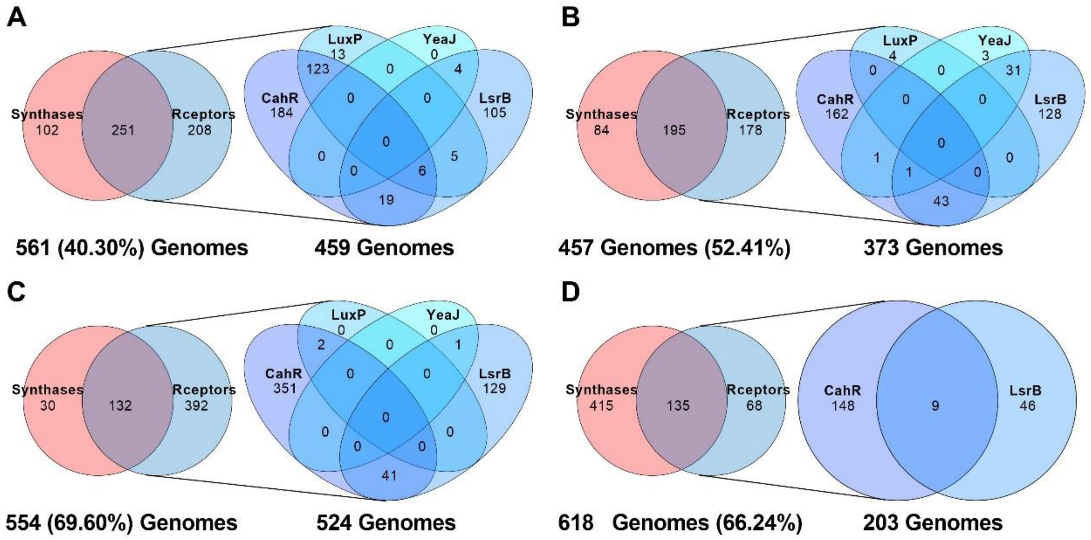

Figure S4. The occurrence of synthases and receptors in four typical habitats. Number of AI-2 synthases and receptors in the ocean **A**, soil **B**, plant rhizosphere **C** and animal gastrointestinal tract **D**, respectively.

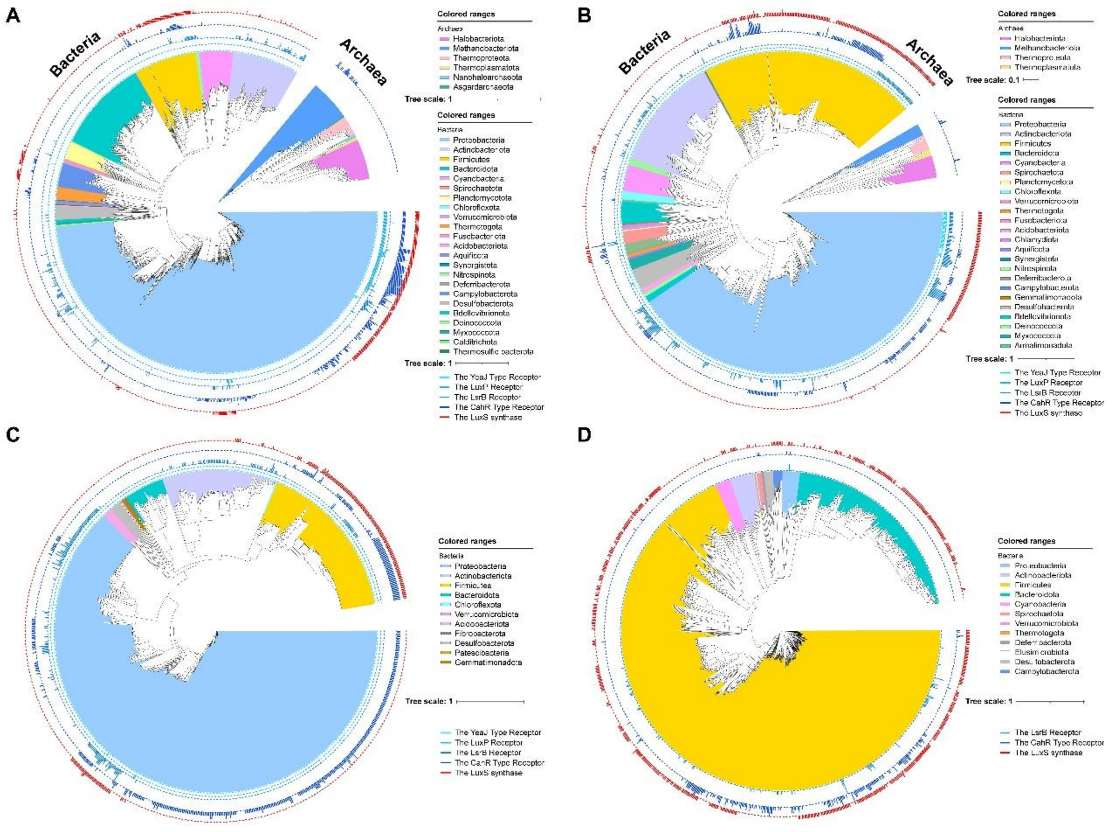

Figure S5. The phylogenetic tree of microbial genomes in four typical habitats. The phylogenetic tree of microbial genomes in the ocean **A**, soil **B**, plant rhizosphere **C** and animal gastrointestinal tract **D**, respectively. The phylogenetic tree was constructed via FastTree v2.1.10 with default parameters according to the protein sequence alignments of 120 core bacterial or 53 core archaeal genes generated by the GTDB-Tk and visualized using iTOL.

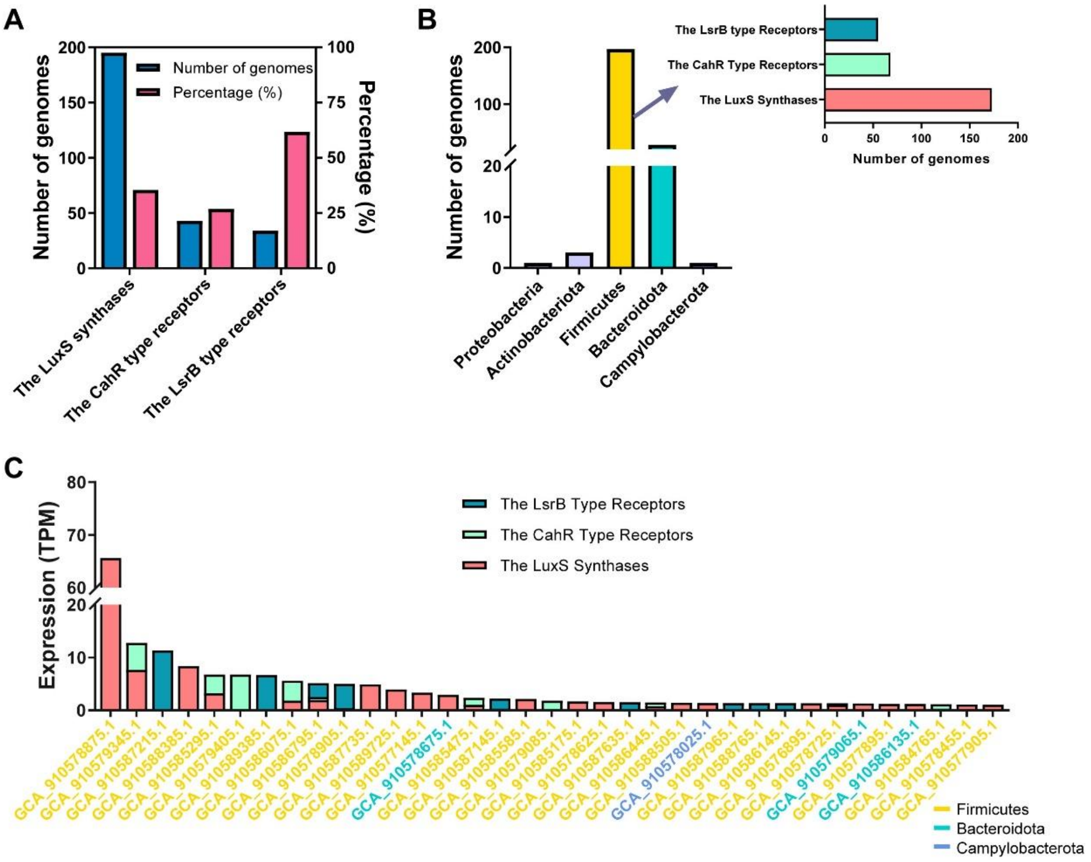

Figure S6. Expression of the identified AI-2-related genes within a mouse gut microbial metatranscriptome. **A** Number of genomes coding AI-2 synthases and receptors. Numbers indicate the numbers of microbial genomes in which the corresponding genes were expressed. The percentage of the corresponding genomes that expressed these genes. **B** The taxa expressed the predicted AI-2 synthases and receptors at the phylum level. **C** Expression (TPM value >1) of the predicted AI-2 synthases and receptors. The colors of the font indicate bacterial phyla.

Figure	Organisms	Protein ID	The binding sites	Number of conserved sites
Figure. 2B	*Streptomyces thermodiastaticus*	WP_189798775.1	KDNQPT	4
	Brevilactibacter sp.	WP_232549263.1	KDNQPT	4
	Microbispora siamensis	WP_193209329.1	KDNQPT	4
	Georgenia thermotolerans	WP_152204434.1	KDNLQT	2
	Fimbriimonas ginsengisoli	WP_025225990.1	KDNQPS	5
	Capsulimonas corticalis	WP_218025743.1	KDNQIS	4
	Atribacter laminatus	WP_218111609.1	KETHSM	1
	Flexilinea flocculi	WP_062279290.1	KESHSM	1
	Chloroflexus islandicus	WP_066785068.1	KDNQPT	4
	Truepera radiovictrix	WP_013177636.1	KDNQLS	4
	Oceanithermus profundus	WP_013457663.1	KDDQPA	6
	Deinococcus psychrotolerans	WP_124869860.1	KDNQPT	4
	Desulfosediminicola flagellatus	WP_167505823.1	KDNQPS	5
	Alteridesulfovibrio inopinatus	WP_034643006.1	KDNQPS	5
	Dictyoglomus thermophilum	WP_012547895.1	KDNQPT	4
	Dictyoglomus turgidum	WP_012582803.1	KDNQPT	4
	Brevibacillus fulvus	WP_239565365.1	RDNQPT	3
	Cohnella thailandensis	WP_185123047.1	KDNQNS	5
	Lacrimispora indolis	WP_024294811.1	KDNQPT	4
	Schlesneria paludicola	WP_040592624.1	KDNQIS	4
	Symmachiella dynata	WP_145377911.1	KDNLFS	3
	Enterovibrio hollisae	WP_005503802.1	KELHYS	2
	Pandoraea terrae	WP_224788802.1	KDNQLV	3
	Oharaeibacter diazotrophicus	WP_126537436.1	KDNQPS	5
	Brachyspira suanatina	WP_048595323.1	KDNLVT	2
	Treponema maltophilum	WP_016524542.1	KEAHFS	2
	Aminobacterium mobile	WP_024822721.1	KELHFS	2
	Dethiosulfovibrio salsuginis	WP_143340864.1	KELHFS	2
	Aminiphilus circumscriptus	WP_026368567.1	KETHSA	2
	Geotoga petraea	WP_135402932.1	KDDQPS	6
	Athalassotoga saccharophila	WP_161847371.1	KDNQIS	4

	Chthoniobacter flavus	WP_006980373.1	KDNMIS	3
	Luteolibacter luteus	WP_169454769.1	KDNQVS	4
Figure. 4B	*Streptomyces coelicolor*	WP_011027125.1	KDNQPT	4
	Streptomyces coelicolor	WP_003978052.1	KDNQPT	4

Table S1. Multiple sequence alignment of the LsrB protein from *Salmonella* and the lsrB-type LBDs of selected proteins.

Amino acid sequence alignment was performed using the ClustalW algorithm embedded in MEGA7 software. Information regarding conserved sites was extracted and presented. The provided table includes, from left to right, the luminescence results, the species names, the protein names, the binding sites, and the number of conserved sites.
